# A Key Role for Inhibins in Dendritic Cell Maturation and Function

**DOI:** 10.1371/journal.pone.0167813

**Published:** 2016-12-09

**Authors:** Roxana Olguín-Alor, Marisol de la Fuente-Granada, Laura C. Bonifaz, Laura Antonio-Herrera, Eduardo A. García-Zepeda, Gloria Soldevila

**Affiliations:** 1 Departamento de Inmunología. Instituto de Investigaciones Biomédicas, Universidad Nacional Autónoma de México, Mexico city, Mexico; 2 Unidad de Investigación Médica en Inmunoquímica. Centro Médico Nacional Siglo XXI, Instituto Mexicano del Seguro Social, Mexico city, Mexico; Oklahoma Medical Research Foundation, UNITED STATES

## Abstract

Inhibins are members of the TGFβ superfamily, which regulate many cellular processes including differentiation, proliferation, survival and apoptosis. Although initially described as hormones regulating the hypothalamus-pituitary-gonadal axis, based on their ability to antagonize Activins, our group has recently reported that they play a role in thymocyte differentiation and survival, as well as in thymic stromal cell maturation and nTreg generation. Here, we used Inhibin knock out mice (Inhα^-/-^) to investigate the role of Inhibins in peripheral dendritic cell maturation and function. We first demonstrated that LPS treated Inhα^+/+^ bone marrow derived dendritic cells (BMDC) were capable to produce significant levels of Inhibin A. Interestingly, Inhα^-/-^ BMDC showed reduced MHCII and CD86 upregulation and increased PD-L1 expression in response to LPS compared to Inhα^+/+^, which correlated with reduced ability to induce proliferation of allogeneic T cells. The “semi-mature” phenotype displayed by Inhα^-/-^ mBMDC correlated with increased levels of IL-10 and slightly decreased IL-6 production after LPS stimulation. In addition, Inhα^-/-^ mBMDC showed impaired migration towards CCL19 and CCL21, assessed by *in vitro* chemotaxis and *in vivo* competitive homing experiments, despite their normal CCR7 expression. Furthermore, *in vivo* LPS-induced DC maturation was also diminished in Inhα^-/-^ mice, specially within the LC (CD207+ CD11b+ CD103-) subpopulation. Finally, analysis of delayed type hypersensitivity responses in Inhα^-/-^ mice, showed reduced ear swelling as a result of reduced cellular infiltration in the skin, correlating with impaired homing of CD207+ DCs to the draining lymph nodes. In summary, our data demonstrate for the first time that Inhibins play a key role in peripheral DC maturation and function, regulating the balance between immunity and tolerance.

## Introduction

Dendritic cells (DC) are a heterogeneous group of professional antigen presenting cells that are critical in the induction and regulation of immune responses. They originate in the bone marrow principally from myeloid progenitors (MP) that undergo differentiation to Macrophage-DC progenitors (MDP), then to Common DC progenitors (CDP) and finally, become Pre-DC that emigrate and seed peripheral tissues where they complete their differentiation to DC [1]. The main cytokines involved in DC differentiation are FLT3, M-CSF and GM-CSF, however, the Pre-DCs are target of the specific-tissue microenvironment. Under steady state conditions, peripheral DCs retain an immature phenotype (iDC) characterized by the low surface expression of MHCII and costimulatory molecules, and by high capacity to sense antigens. When iDCs capture an antigen in the presence of a pro-inflammatory microenvironment, they enter a maturation process. As a result, DC become mature and capable to activate T cells due to high expression of MHCII and costimulatory molecules and the secretion of pro-inflammatory cytokines such as IL-1β, IL-6, IL-12p40 and p70, among others. Otherwise, if the microenvironment lacks pro-inflammatory cytokines or in the presence of an anti-inflammatory milieu, the iDC becomes a tolerogenic DC leading to T cell anergy, T cell deletion and/or induction of regulatory T cells (Tregs) (reviewed in [2–4]). Members of the TGFβ superfamily, such as Transforming Growth Factor β (TGFβ), Activins and bone morphogenetic proteins (BMPs,) have been shown to influence these processes, (reviewed in [5]).

The TGFβ superfamily comprises soluble mediators that regulate cellular processes including differentiation, proliferation and apoptosis (reviewed in [6]). There is growing evidence on the role of TGFβs, Activins and BMPs, as regulators of the immune system (reviewed in [7]), however very scarce information is available about the role of Inhibins. These are glycoprotein hormones that down-regulate follicle-stimulating hormone (FSH) production by the anterior pituitary whereas their counterparts, the Activins, up-regulate FSH production. Structurally, Inhibins are composed by two subunits, an α- and a β-subunit, linked by a disulfide bridge, while Activins are homodimers of β-subunits (reviewed in [8,9]). The signaling pathway for members of TGFβ superfamily is shared among TGFβ, BMPs and Activins, in which the dimeric ligands bind type I and type II receptors with serine/threonine kinase activity, leading to the phosphorylation of cytoplasmic proteins known as receptor SMADs, which then heterodimerize with the common SMAD (co-SMAD) and translocate to the nucleus to regulate gene expression (reviewed in [8]). In contrast, Inhibins bind type II receptors through their β-subunit and, instead of type I receptors, bind betaglycan (BG), a non-signaling type III TGFβ receptor (TβRIII), through their α subunit, thereby excluding the type I receptor (ALK4) from the ternary receptor complex (reviewed in [8]). Thus, Inhibins were considered as ligands unable to induce signal transduction and through this mechanism they may antagonize Activin functions. However, several data have suggested the possibility that Inhibins may act through an independent receptor, although, an Inhibin-specific binding molecule has not yet been identified (reviewed in [9]).

Although initial findings described the antagonistic role of Inhibins on Activin-mediated functions, there is growing evidence demonstrating that Inhibins can mediate functions on different cell types, independently of Activins (reviewed in [10]). In this context, our group has demonstrated that Inhibins play a role in regulating specific checkpoints during thymocyte differentiation and shown that Inhibins do not always antagonize Activin functions during T cell development [11]. Moreover, we have recently reported that Inhibins play a role in controlling thymic stromal cell differentiation and maturation. In this context, the absence of Inhibins promoted medullary over cortical Thymic Epithelial Cell (TEC) differentiation and reduction of MHCII and CD86 expression on thymic conventional DCs (cDCs). [12].

Although some reports have demonstrated the role of Activins in DC maturation (reviewed in [13]), the specific role of Inhibins in DCs remains elusive. In addition, these ligands may exert different effects depending on the DC subset, maturation stimulus and tissue microenvironment. For this reason, here we investigated the role of Inhibins in DC maturation and function.

## Materials and Methods

### Mice

Wild type (Inhα^+/+^) and Inhibin α-deficient (Inhα^-/-^) mice in C57BL/6 background, were generated and kindly gifted by Dr. Martin Matzuk (Baylor College of Medicine, Houston TX) [14]. Actin-green fluorescent protein (GFP) transgenic mice on the C57BL/6 background were obtained from Dr M. Okawa [15] (Genome Information Research Center, Osaka University, Japan). Foxp3^EGFP^ *knock in* mice on the BALB/c background (Cg-Foxp3tm2Tch/J) were purchased from Jackson Laboratories. All mice were bread and maintained in SPF conditions in the animal facility of the Instituto de Investigaciones Biomédicas (IIB, UNAM, México) according to ethics guidelines. The study was approved by the "Comité para el Cuidado y Uso de Animales de Laboratorio (CICUAL)” of the IIB.

### Generation of bone marrow-derived DC

Bone marrow cells were obtained from femurs and tibias of 9-week-old Inhα^+/+^ and Inhα^-/-^ female mice as previously described [16]. The cells were resuspended in RMPI 1640 supplemented with 10% heat inactivated FCS, 100 U/ml penicillin, 100μg/mL streptomycin and immature bone marrow derived DC were differentiated in the presence of 5% supernatant of a GM-CSF producing cell line [17]. For DC maturation, on day 5, iBMDC were stimulated with 1μg/ml E. coli 0111:B4 LPS (Sigma Aldrich) for 24 hours (mBMDC). On day 6, non-adherent cells (containing 50–60% of CD11c+ MHCII+ DC) were harvested and used for *in vitro* and *in vivo* experiments.

### Preparation of DCs from peripheral lymphoid organs

Spleen (SP), and periphery lymph nodes (LN) from 3-week-old Inhα^+/+^ and Inhα^-/-^ female mice were chop into small pieces and digested with 5mg/ml of type IV Collagenase (GIBCO) and 25 U/ml DNAse (Roche) in RPM 1640 supplemented with 5% heat inactivated FBS for 1 hour at 37°C. Subsequently, the remaining tissue was mechanically disaggregated. Cell suspensions were filtered using a 50μm mesh and washed with PBS. For the spleen, erythrocytes were lysed with ACK buffer during 2 minutes and washed with PBS. Finally, cells were resuspended in FACS buffer for flow cytometry analysis.

### Preparation of DCs from skin

Ears from 3 week-old Inhα^+/+^ and Inhα^-/-^ mice were cut off at the base and separated into dorsal and ventral halves. Skin tissue was digested with 0.25mg/ml Liberase TL (Roche) and 0.125mg/ml DNAse grade II (Roche) in RPMI 1640 media for 45 minutes at 37°C. Subsequently, the skin was cut into small pieces and incubated for an extra 45 minutes at 37°C. Then, the digestion was stopped with 5μM of EDTA in RPMI 1640 supplemented with 10% FBS. Cells were resuspended in 0.125mg/ml DNAse RPMI 1640 10% FBS and incubated 5 minutes on ice. Finally, cells were washed and resupended in RPMI 1640 10% FBS for flow cytometry analysis.

### Flow cytometry

For phenotypic analysis cells were incubated with primary antibodies for 20 min at 4°C, followed by two washes with FACS Buffer. When necessary, fluorochrome-coupled streptavidin was added, and incubated for 20 min at 4°C and washed. Cells were fixed with 4% paraformaldehyde.

The BMDC were stained with anti-I-A/I-E Alexa Fluor 488 (M5/114.15.2) and anti-CD80 PerCp/Cy5.5 (16-10A1) from Biolegend (San Diego, CA); anti-CD11c PE (N418), anti-CD11c PE Cy7 (N418) and anti-CD86 APC (GL-1) from Tonbo Biosciences (San Diego, CA); anti-CD40 PE (3/23), anti-CD274 PE (MIH5) and anti-CD273 PE (TY25) from BD Biosciences (San Jose, CA); a-TLR4/MD2 complex PE Cy7 (MTS510) and a-CD197 PerCp/Cy5.5 from eBiosciences (San Diego, CA).

For DC subpopulation staining *ex vivo* and after *in vitro* LPS stimulation, the cells were previously blocked with purified anti-CD16/32 (93), followed by staining with anti-I-A/I-E Alexa Fluor 488 (M5/114.15.2) and anti-CD11c Alexa Fluor 700 (N418) from Biolegend, anti-CD3 PE (145-2C11), anti-TER119 PE (TER-119), anti-CD8α APC (53–6.7), anti-CD11b Violet Fluor 450 (M1/70) and anti-CD11c PE Cy7 (N418) from Tonbo Biosciences, anti-CD19 PE (1D3), anti-NK PE (2B4) and Streptavidin APC Cy7 from BD Biosciences; anti-CD103 biotin (2E7), anti-CD207 (eBioRMUL2) from eBiosciences and anti-rat IgG Qdot 605 from Invitrogen (Eugene, OR).

All samples were acquired in an Attune Acoustic Focusing Flow Cytometer (Life Technologies) and were analyzed using FlowJo 10 software (Tree Star Inc.).

### Immunofluorescence and microscopy

BMDC. On day 5 of culture, the CD11c^+^ cells were enriched by positive selection using anti-CD11c micro beads from Miltenyi Biotec and placed onto glass coverslips treated with fibronectin from Calbiochem for 24 hours with or without LPS (1μg/ml). The cells were fixed in 3.7% paraformaldehyde and permeabilized with 0.1%. Triton-X100. Then, cells were blocked with 1% Bovine Serum Albumin (BSA) in PBS and stained with anti-I-A/I-E Alexa Fluor 488 and Rhodamine-phalloidin. Coverslips were mounted with fluorescence mounting medium from DAKO (Denmark A/S). Cells were observed with a Zeiss LSM5 confocal microscope equipped with LSM5 PASCAL 2.8 software and image analysis of cell area based on Rhodamine-phalloidin staining was performed with ImageJ 1.46r software. A cell density that precluded the formation of cellular aggregates and allowed an accurate quantification of individual cells was used. Single cell analysis was performed from three independent experiments giving an *n* value of 60 per condition. Data were expressed as percent of cell spreading, normalized to mean value of Inhα^+/+^ iBMDC cells (100%).

Epidermal sheets were obtained from ears of 3-week-old Inhα^+/+^ and Inhα^-/-^ female mice. Ears were cut off at the base and the skin was separated into epidermis and dermis by using 5 mM EDTA incubation for 1 hour at 37°C. The epidermal sheets were rinsed with PBS, fixed in cold acetone, blocked with 1% BSA in PBS and stained with anti-I-A/I-E Alexa Fluor 488 and anti-CD80 Per CP/Cy5.5. Finally, epidermal sheets were mounted with Dako fluorescence mounting medium. Sheets were observed with a Zeiss LSM5 confocal microscope and an Olympus IX71 inverted microscope. Analysis of cell counts and fluorescence intensity was performed with ImageJ 1.16r software. At least three independent experiments were performed giving an *n* value of 60 micrographs using a 40x objective for cell counts. For *in vivo* maturation, single cell analysis was performed from each condition acquiring an average of 15 confocal micrographs using a 100x oil immersion objective and quantifying an average of 75 cells per condition. Data were obtained calculating the ratio between the mean fluorescence intensity (MFI) of stimulated cell divided by the MFI of non-stimulated cells and was expressed as relative increment (RI).

### Chemotaxis assays

Chemotaxis was perfomed as previosulsy described [18]. Briefly Inmature BMDC (iBMDC) and LPS-stimulated BMDC (mBMDC) were fluorescently labelled with calcein-AM from Molecular Probes (Eugene, OR) and resuspended in Hank’s balanced salt solution (HBSS) 0.5% BSA at 2 x 10^6^ cells/ml. Chemotaxis assays were performed using a 48-well Boyden chamber, increasing concentrations (0–1000 ng/ml) of CCL19 and CCL21 chemokines were placed in the lower wells of the chamber and in the upper wells 1 x 10^5^ cells allowed to migrate through fibronectin-coated 5μm pore polycarbonate membranes from Neuroprobe (Gaithersburg, MD). Chemotaxis index was calculated by dividing the fluorescence of chemokine stimulated cells by the fluorescence of wells with media alone (chemokinesis). After 1.5 hours, trans-migrated cells were quantified using a Typhoon Scanner 9400.

### Calcium mobilization

2.5 × 10^6^ cells/ml were resuspended in RPMI-1640 2% of FBS and stained with 4.59 μM Fura-Red and 2.6μM Fluo-3 (Molecular Probes) for 45 minutes at 45 min at 37°C in the dark. Cells were washed twice in RPMI 2% FBS and incubated for an extra 45 min at room temperature. Then cells were washed and resuspended in 1ml RPMI 0% FBS prior FACS analysis. Cells were acquired for 100s for baseline Ca++ and then stimulated with pre-warmed CCL19 and CCL21 diluted in RPMI 0% FBS 300s. As positive control for dye loading samples were stimulated with PMA (40ng/ml) and Ionomycin (400ng/ml) The data were analyzed with FCS Express 5 Plus software (De Novo software) and expressed as the median Ca^2 +^ fluorescence ratio (FL1H/FL3H).

### Erk phosphorylation

Phosphorylation of Erk was detected by flow cytometry after intracellular staining following a slightly modified phospho-flow (BD Biosciences) Briefly, 10^6^ mBMDC were pre-stained with MHC II and CD11c antibodies and then stimulated with chemokines (300ng/ml CCL19 and CCL21) in RPMI 1640 medium supplemented with 0.2% SFB at 37°C for 30s, 1min, 3min and 5min. Activation was arrested by fixation with 150 μL of Lyse/Fix buffer for 10 min at 37°C. To permeabilize, cells were incubated with 100 μL Perm buffer II for 20 min at 4°C, then washed and stained with PE-CF594 anti-pErk antibody (Biolegend). All data were analyzed with FlowJo 10 Tree Star software. Relative increment of pErk was calculated as MFI of stimulated cells/MFI of unstimulated cells.

### *In vivo* migration of BMDC

*In vivo* migration assays were performed as previously described [19]. Briefly, LPS-stimulated BMDC from Actin GFP *knock in* mice (Inhα ^+/+^) and BMDC from Inhα ^-/-^ mice stained with Cell Trace Violet (CTV) (Life Technologies), according to manufacturer’s instructions, were mixed 1:1 in sterile PBS. Viability of labeled cells previous to and after the *in vivo* transfer was 85–90%. A total of 2 x 10^6^ cells in 50μl were injected in left footpad and, as a control, 50μl of PBS in right footpad of 6–9 week-old female C57BL/6 mice. After 48 hours, popliteal lymph nodes were extracted and stained with viability dye Aqua Zombie (Invitrogen) and anti-CD11c PE (Tonbo).

### Cytokine quantification

Supernatants from BMDC cultures stimulated with LPS, LPS + Inhibin A or with media alone were collected 3, 6, 12, 18 and 24 hours after stimulation and stored at -20°C until use. IL-6, IL-10, TNF and CCL2 were quantified with the CBA kit of murine inflammatory cytokines (BD Biosciences, San Jose, CA) following the instructions of the manufacturer. Samples were acquired in a FACS Calibur cytometer from BD Biosciences and analyzed with FCAP Array software. Activin A and Inhibin A were quantified using the Human/Mouse/Rat Activin A Quantikine ELISA Kit (R&D systems) and Mouse InhA ELISA Kit (Elabscience) following the instructions of the manufacturer.

### Proliferation assays

CD4^+^CD25^-^Foxp3^-^ T cells from spleen and peripheral LN from Foxp3 GFP *knock in* female mice were sorted by flow cytometry (FACS Aria I, Becton & Dickinson) and labelled with CTV. 1.25 x 10^5^ T cells were seeded into 96 well plates. CD11c microbead- enriched BMDCs were co-cultured with CD4^+^CD25^-^Foxp3^-^ T cells at 1:1, 1:2, 1:4; 1:10 and 1:20 ratios. After 3 and 5 days of culture, cells were stained with anti-CD4 PE (Tonbo) and Zombie Aqua (Biolegend). Proliferation was evaluated by CTV dilution within the live (zombie aqua^-^) CD4^+^ cells and calculated as % of divided cells, using the formula:

$$\%\text{ of divided cells }=\;\frac{\#\text{ cells that went into division}}{\#\text{ cells at start of culture}}\text{ x }100$$

### *In vivo* LPS stimulation of DCs

Three-week-old female mice were inoculated with 1μg of LPS in the ear (left), as a control PBS (right) was inoculated in the opposite ear. After 6, 18 and 72 hours, MHCII, CD80, CD86 and PD-L1 were analyzed in DCs from the draining lymph node (dLN) by flow cytometry, as well as MHCII and CD80 in LCs from epidermal layers of the ear by immunofluorescence microscopy as describe above.

### Delayed Type Hypersensitivity (DTH)

Three week-old female mice were immunized with 100ng of ovalbumin (OVA) and 10μg of LPS subcutaneously in the back. Control mice were inoculated with PBS, 100ng of OVA or 10μg of LPS. Seven days later mice were challenged with 100ng OVA in one ear, and PBS in the other. After 24 hours, ear thickness was measured using an electronic digital micrometer and expressed as relative increment of the OVA challenged ear compared to PBS. Whole ears were embedded in paraffin, and sections taken for histochemical analysis, using Hematoxylin-Eosin staining. Twenty-seven fields from each ear section were analyzed for cellular infiltration and expressed as cells/mm^2^.

### Statistical analysis

Data are presented as mean values ± SEM. The significance of results was calculated by paired or unpaired, one or two-tailed Student T-test, utilizing GraphPad Prism 6 statistical software. *P* values <0.05 was considered as statistically significant. *p<0.05, **p<0.01, ***p<0.001.

## Results

### Impaired *in vitro* DC differentiation and maturation in the absence of Inhibins

**Fig 1:**
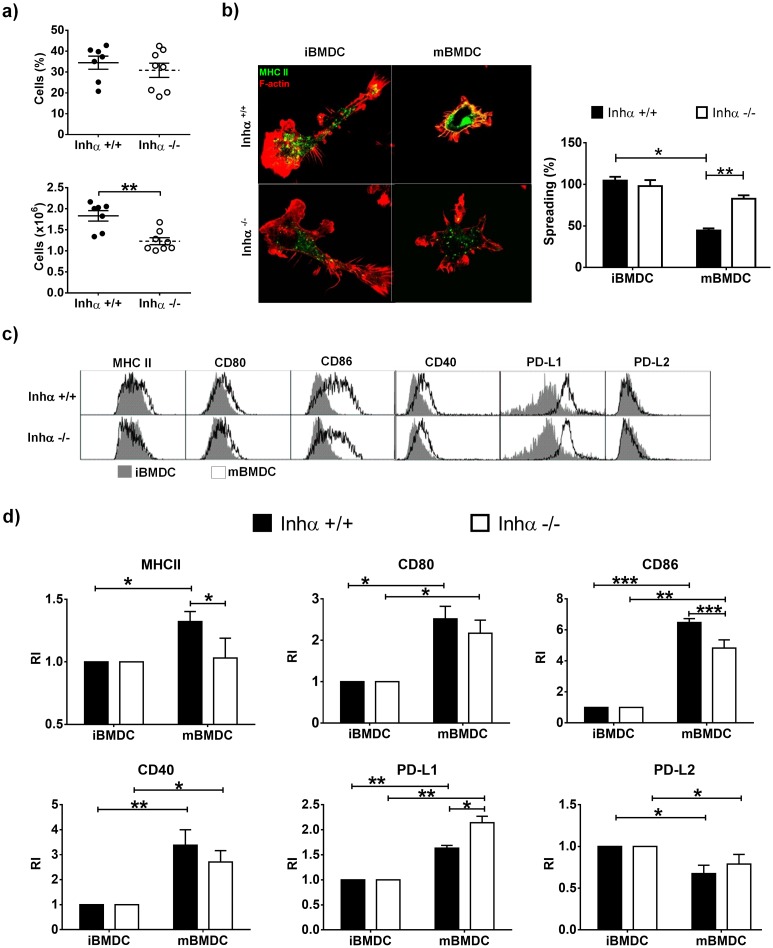
Impaired differentiation and maturation of Inhibin α ^-/-^ BMDC after LPS stimulation. DCs were derived *in vitro* from bone marrow progenitors of Inhα^+/+^ or Inhα^-/-^ mice in the presence of GM-CSF. A. At day 6, BMDCs were harvested and percentages (top) and cell numbers (bottom) of DC (CD11c^+^ MHCII^+^) were analyzed by flow cytometry. Graphs represent the mean ± SEM of 4 independent experiments (n = 8). B. BMDC were stained for F-actin (red) and MHCII (green) in a fibronectin-treated surface for morphology analysis by immunofluorescence. Bar graphs represent the mean ± SEM of 3 independent experiments. C-D. At day 5 of culture, the cells were stimulated with LPS (1μg/mL) to induce maturation. After 24h, BMDCs were harvested and expression of MHCII, CD40, CD80, CD86, PD-L1 and PD-L2 was analyzed by flow cytometry gated on DC population. Representative histograms of Inhα^+/+^ (top) or Inhα^-/-^ BMDC (bottom) (C). MFI was determined and reported as relative expression compared to Inhα^+/+^ mice. Bar graphs show the mean ± SEM of 4 independent experiments (D). Asterisks denote statistical significance *p≤0.05; **p≤0.01; ***p≤0.001.

We have previously demonstrated that in the absence of Inhibins, thymic cDCs display reduced levels of MHCII and CD86 [20]. On the other hand, it has been suggested that Activin A and Inhibin A, can promote tolerogenic DCs [21]. To investigate the role of Inhibins in DC biology, DCs were generated *in vitro* from BM progenitors from Inhα^-/-^ and Inhα^+/+^ mice in the presence of GM-CSF (immature BMDC: iBMDC), followed by 24h LPS stimulation to induce maturation (mature BMDC: mBMDC). Interestingly, although the percentage of DCs in the culture was not altered in the absence of Inhibins, the absolute number of DCs was significantly reduced compared to Inhα^+/+^ (Fig 1A). This reduction in DC numbers was not due to increased cell death in any of the subpopulations analyzed, as shown by Annexin V and 7-AAD staining (S1A and S1B Fig).

Immature DCs, characterized by an extended and adherent phenotype and presence of podosomes, change to a rounded and non-adherent migratory phenotype after maturation [22]. These morphological changes are accompanied by an increased surface expression of MHC Class II (MHCII) and co-stimulatory molecules such as, CD80, CD86, and CD40, among others [23]. As expected, Inhα^+/+^ BMDC decreased their spreading when matured with LPS but in contrast, Inhα^-/-^ LPS-stimulated BMDC, retained their spreaded morphology, resembling immature DCs (Fig 1B and S1C Fig). Furthermore, MHCII and CD86 levels were significantly diminished in Inhα^-/-^ mBMDC compared to Inhα^+/+^ mBMDC, and CD80 and CD40 also exhibited a trend towards a decrease, while PD-L1 showed a significant increased expression (Fig 1C). The impaired upregulation of the co-stimulatory molecules was not due to a reduction in TLR4 expression on the surface of Inhα^-/-^ BMDC (S1D Fig). Interestingly, a slight decrease in MHCII upregulation was found in Inhα^-/-^ iBMDC in response to anti-CD40 and Poly I:C, suggesting that impaired maturation may be the result of an intrinsic defect in DC maturation rather than TLR4 deficient signaling pathway (S1E Fig).

### CCR7 dependent migration is impaired in Inhα^-/-^ mBMDC

**Fig 2:**
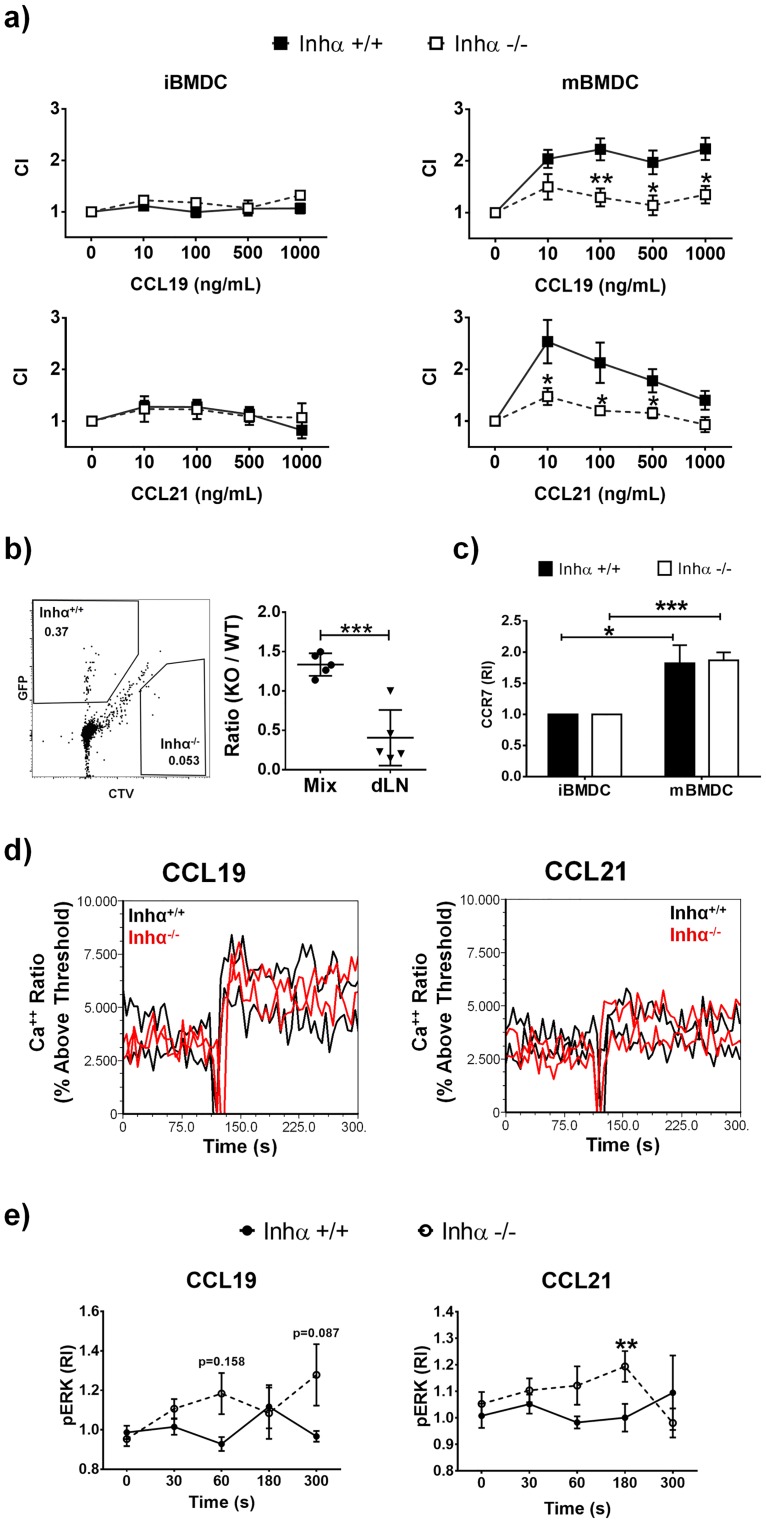
LPS-stimulated Inhα^-/-^ BMDC have impaired migration towards CCL19 and CCL21. A. Chemotaxis towards CCL19 and CCL21 were performed with Inhα^+/+^ and Inhα^-/-^ unstimulated or 24h LPS-stimulated BMDCs. Graphs show the chemotaxis index expressed as mean ± SEM (n = 4 independent experiments). B. Mix of Inhα^+/+^ (GFP^+^) and Inhα^-/-^ (CTV^+^) BMDC (1:1) were inoculated in a C57BL/6 footpad and 48 hours later dLN were analyzed for the presence of BMDC. Graph represents the ratio of the mix inoculated and the ratio obtained in the dLN (mean ± SEM of 2 independent experiments; n = 5). C. CCR7 surface expression in BMDC. MFI was determined and reported as relative expression compared to Inhα^+/+^ mice. Bar graphs show mean ± SEM of 3 independent experiments. D) Calcium flux analysis of Inhα^+/+^ and Inhα^-/-^ mBMDC stimulated with 300ng/ml of CCL19 and CCL21. E) Time course of Erk phosphorylation in response to CCR7 ligands in Inhα^+/+^ and Inhα^-/-^ mBMDC analyzed by flow cytometry. MFI was determined and reported as relative expression compared to unstimulated cells. Statistical significance was determined by a two-tailed unpaired Student t-Test. *p≤0.05; **p≤0.01.

During DC maturation, the morphological changes to a round non-adherent cells combined with the upregulation of the chemokine receptor CCR7 allows a rapid migration of DCs to the lymph node, were naïve T cell activation takes place. As Inhα^-/-^ DCs displayed an impaired maturation, we next evaluated the migration capacity of BMDC using *in vitro* chemotaxis assays towards CCL19 and CCL21, both CCR7 ligands. As expected, Inhα^+/+^ mBMDC showed efficient migration to both chemokines at concentrations ranging between 10 to 1000 ng/mL for CCL19 (Fig 2A, upper panels) and 10 to 500 ng/mL for CCL21 (Fig 2A, lower panels), in contrast to their immature counterparts, that migrated poorly towards these chemokines. In contrast, mBMDC from Inhα^-/-^ mice were unable to migrate towards CCL19 or CCL21, similarly to iBMDC (Fig 2A). Furthermore, *in vivo* migration assays where a mixture of Inhα^+/+^ (GFP^+^) and Inhα^-/-^ (CTV^+^) mBMDCs were injected into the footpad of C57BL/6 mice, showed that Inhα^-/-^ mBMDC migrated poorly to the dLN evaluated as early as 4 hours after injection (data not shown) and up to 48 hours in comparison with Inhα^+/+^ BMDCs (Fig 2B). To ensure that the staining process did not affect the viability or migration ability of Inhα^-/-^ BMDC, parallel assays were performed using Inhα^+/+^ BMDC and Inhα^-/-^ BMDC stained with CTV and CFSE, respectively, showing similar results (data not shown). This impaired migration was not explained by CCR7 expression, as there were no significant differences in CCR7 surface levels between Inhα^+/+^ and Inhα^-/-^ mBMDCs (Fig 2C). In order to investigate the molecular mechanisms underlying the impaired migration of Inhα^-/-^ mBMDCs, we analyzed Ca++ mobilization (Fig 2D) and Erk phosphorylation (Fig 2E) in response CCR7 ligands. Although Ca^++^ fluxes induced by CCL19 and CCL21 did not show significant differences between Inhα^+/+^ and Inhα^-/-^ BMDCs, unexpectedly, Erk phosphorylation was increased significantly increased in Inhα^-/-^ BMDCs at different time points after chemokine stimulation.

### mBMDC from Inhα^-/-^ mice show impaired allogeneic T cell stimulation capacity

**Fig 3:**
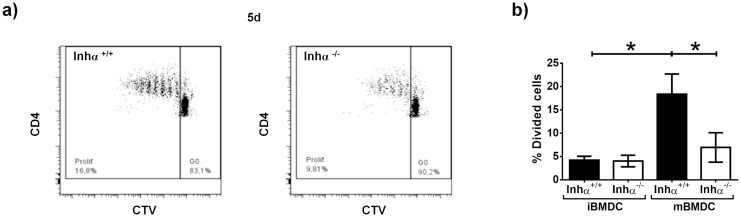
BMDC Inhα^-/-^ showed poor allogeneic CD4^+^ CD25^-^ Foxp3^-^ T cells stimulation capacity. **Unstimulated and 24h LPS-stimulated** CD11c^+^ cells were sorted at day 6 of culture and were co-cultivated with CD4^+^ CD25^-^ Foxp3^-^ T cells from Foxp3-GFP KI Balb/c. At day 5, proliferation of T cells was measured by CTV dilution. A. Representative dot plot of CTV dilution from Inhα^+/+^ BMDC (left) and Inhα^-/-^ BMDC (right). B. Graphs of divided T cells at 1:10 ratio represent mean ± SEM of 4 independent experiments. Statistical significance was determined by a two-tailed unpaired Student t-Test. *p≤0.05.

A characteristic of fully mature DC is their ability to activate naïve T lymphocytes [24]. Since Inhα^-/-^ mBMDCs appeared to display a “semi-mature” phenotype, we next evaluated their ability to activate allogeneic CD4^+^. T cells. Immature or LPS-stimulated CD11c^+^ BMDC were co-cultured with allogeneic CD4^+^CD25^-^Foxp3^-^ T cells pre-labelled with CTV and after 3 (not shown) and 5 days, proliferation was assessed by CTV dilution on live CD4^+^ gated T cells. As shown in Fig 3, both Inhα^+/+^ and Inhα^-/-^ iBMDCs induced a negligible T cell proliferation at 5 days. Inhα^+/+^ mBMDC induced significant T cell proliferation compared to their iBMDCs counterparts. Interestingly, Inhα^-/-^ mBMDC did not induce a significant increase in T cell proliferation compared to Inhα ^-/-^ iBMDCs and was significantly lower than Inhα^+/+^ mBMDC (ratio 1:10, Fig 3B).

### mBMDC from Inhα^-/-^ mice produce increased IL-10 levels in response to LPS

**Fig 4:**
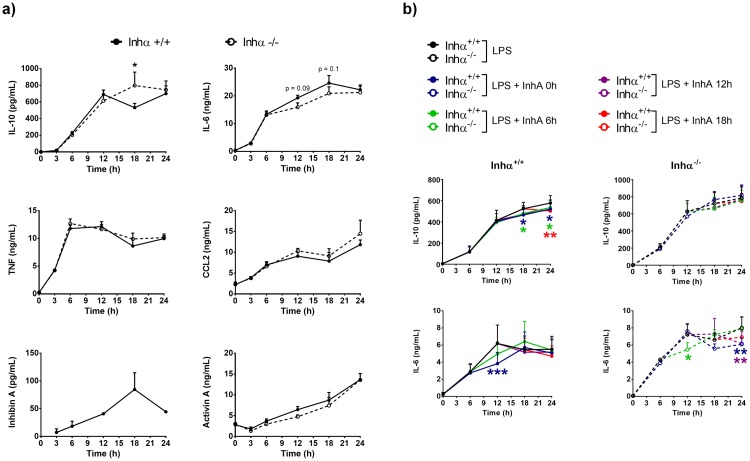
Inhα^-/-^ BMDC produce increased IL-10 after LPS stimulation. A) Time course of cytokines from supernatants of BMDC cultures were quantified by ELISA (Inhibin A and Activin A) and CBA (IL-6, IL-10, TNF and CCL2). Graphs represent mean ± SEM of at least 3 independent experiments and 2 independent experiments for Inhibin A. b) Time course of IL-10 and IL-6 production by Inhα^+/+^ (left) and Inhα^-/-^ (right) BMDCs in the presence of exogenous recombinant Inhibin A (150pg/ml) at 0h, 6h, 12h and 18h post stimulation with LPS. Data represent the mean ± SEM of 3 independent experiments (n = 3 mice). Statistical significance was determined by using a two-tailed unpaired Student t-Test *p≤0.05; **p≤0.01; ***p≤0.001.

Since Inhα^-/-^ BMDC maturation was altered, we further investigated whether the absence of Inhibins affected their ability to secrete pro-inflammatory cytokines, such as IL-1β, IL-2, IL-12, IL-6, which are associated with a proper T cell activation [23], as well as the anti-inflammatory cytokines IL-10 and TGFβ which are associated to tolerance induction [25] We evaluated the production of TNF, IL-6, IL-12, CCL2, IFNγ and IL-10 by CBA before and 3, 6, 12, 18 and 24 h after LPS stimulation in BMDC culture supernatants. IL-12 and IFNγ were undetectable at all time points (data not shown). As shown in Fig 4, TNF and CCL2 were similarly produced by Inhα^-/-^ and Inhα^+/+^ mBMDC. Interestingly, Inhα^-/-^ mBMDC displayed significantly increased levels of IL-10, and a trend towards a decrease in IL-6 production at 18h after LPS stimulation, reaching the levels of Inhα^+/+^ BMDC at 24 hours. Although TGFβ was not detected in the BMDC cultures at any time point (data not shown) the disparity between down modulation of maturation markers and production of inflammatory cytokines resembles the “semi-mature” phenotype previously reported in tumor associated DCs which have been associated with anergy, Treg induction and impaired anti-tumor immunity [26].

A previous report showed that recombinant Activin A and Inhibin A can down modulate DC maturation *in vitro* [27]. In addition, it has been previously demonstrated that monocyte-derived DCs produce large amounts of Activin A after TLR stimulation, as early as 2 hours [28], while there are no reports on Inhibin production by these cells. Therefore, we investigated whether Activin A and Inhibin A could be produced during LPS-mediated BMDC maturation. Our results show that Activin A is produced after 6h of LPS stimulation and further increases up to 24h hours, while Inhibin A is secreted by BMDC after 6 hours of LPS stimulation, having a maximum concentration at 18 hours, which decreased at 24 hours (Fig 4). These data suggested that Inhibin A might act on BMDC in an autocrine manner. Interestingly, both Inhα^+/+^ and Inhα^-/-^ BMDC secreted equal amounts of Activin A, arguing against the possibility that the impaired maturation observed in Inhα^-/-^ BMDC was due to increased Activin A production. Interestingly, addition of exogenous recombinant Inhibin A during LPS stimulation (0h, 6h, 12 h and 18h post-LPS treatment) led to a reduction of IL-6 production by both Inhα^+/+^ and Inhα^-/-^ BMDC, and a decrease in IL-10 production in Inhα^+/+^ BMDC, in agreement with data previously reported by Segerer et al. [27].

### Inhα^-/-^ mice have normal percentages and numbers of DC subsets under homeostatic conditions

**Fig 5:**
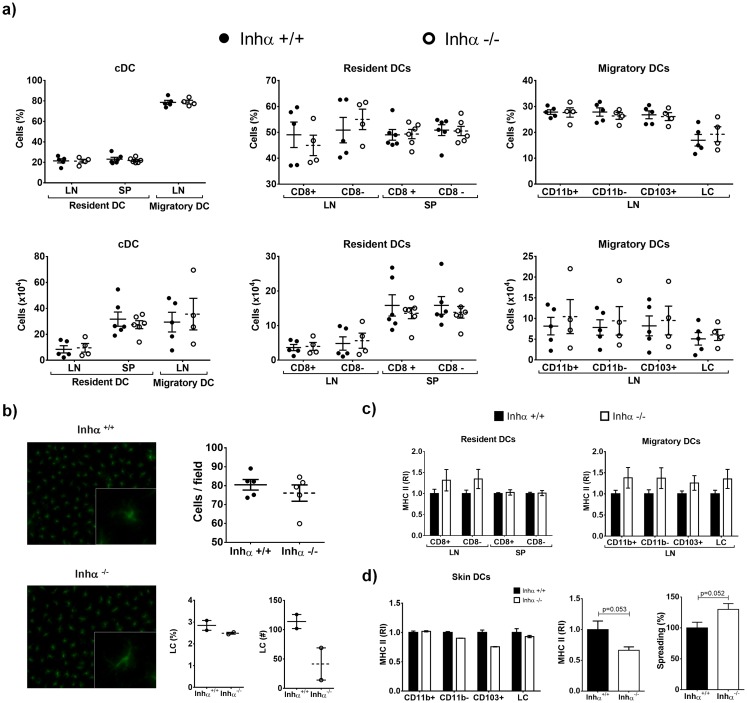
DCs percentages and numbers *in vivo* are not affected in absence of Inhibin α, but LC have low MHCII expression. Inhα^+/+^ and Inhα^-/-^ 3 week-old mice were analyzed for cDCs (Lin^-^ CD11c^+^ MHCII^+^), migratory DCs (Lin^-^ CD11c^+^ MHCII^hi^) and resident DCs (Lin^-^ CD11c^hi^ MHCII^+^) in lymph nodes and spleen by flow cytometry. Right graph: further characterization of migratory LN subpopulations based on CD11b, CD103 and CD207 expression as described in S2 Fig. A. Graphs represent frequency (top) and total numbers (bottom) (mean ± SEM) of at least 3 independent experiments (n = 4–6 mice). Subpopulation analysis of resident and migratory DC was performed using the gate strategy described in S2 Fig. B. Ear epidermal layers were stained with MHCII Alexa Fluor 488 for LC analysis by immunofluorescence. Representative images at 20x (left) and graphs represent mean ± SEM of 4 independent experiments expressed as cells/field. Lower panels show FACS analysis of LC (CD207+, CD11b+, CD103-) obtained after digestion of skin sheets under steady state conditions (left, percentages; right, total numbers). C. Analysis of MHCII expression from LN and SP DC subpopulations. Bar graphs represent relative expression of MHCII MFI in DC subpopulations compared to Inhα^+/+^ mice (mean ± SEM). D. Analysis of MHCII expression in skin DCs. Left: FACS analysis of MHCII surface expression (n = 2). Middle: Immunofluorescence analysis of MHCII in epidermal sheets. Data are expressed as relative increment compared to Inhα^+/+^. Right: Analysis of cell spreading in LCs from epidermal layers of Inhα^-/-^ mice compared to Inhα^+/+^. Statistical significance was determined by using a two-tailed unpaired Student t-Test.

The impaired DC differentiation observed *in vitro* (Fig 1) led us to investigate whether the absence of Inhibins may also affect DC differentiation *in vivo*. For this, we evaluated different DC subsets within LN, SP and skin of 3-week-old Inhα^-/-^ mice in homeostasis (S2 Fig), as it was reported that these mice have increased Activin A levels in peripheral blood and develop gonadal tumors after 4 weeks of age [14]. As shown in S2A Fig, at the time of these analyses levels of serum Activin A were not significantly increased compared to controls. We analyzed cDC as resident (Lin^-^ CD11c^hi^ MHCII^lo^) and migratory (Lin^-^ CD11c^lo^ MHCII^hi^) in LN and SP (S2B and S2C Figs), as previously reported by Idoyaga et al [29], and found no differences in numbers and percentages between Inhα^-/-^ and Inhα^+/+^ (Fig 5A). It has been reported that DC subsets can differentially shape the immune responses; for instance, CD8α^+^ DCs and CD103^+^ DCs are specialized to induce a tolerogenic response under certain conditions, compared to their counterparts, CD8α^-^ DCs and CD103^-^ DCs [29]. Analysis of these DC subpopulations showed no differences in the percentage or numbers of CD8α+ and CD8α- resident DCs subset. In order to characterize migratory DCs CD207, CD11b and CD103 markers were used following the gating strategy shown in S2 Fig. As shown in Fig 5A neither the percentage nor the total cell numbers of CD11b+, CD11b-, CD103+ or LC (CD207+ CD11b+ CD103-) subsets were significantly different between Inhα^+/+^ and Inhα^-/-^ mice (Fig 5A).

Since Activin A was shown to promote Langerhans cell (LC) differentiation from monocytes in the skin [30,31] and Inhα^-/-^ mice showed increased Activin A serum levels (S2A Fig and [14]), we also investigated whether the absence of Inhibins could impact the LC differentiation *in vivo*. However, as shown in Fig 5B, there were no differences in the numbers of LC in Inhα^-/-^ mice compared to Inhα^+/+^ mice. This result was further confirmed after digestion of skin sheets and analysis by flow cytometry (Fig 5B bottom panels). We have previously shown that thymic cDC from Inhα^-/-^ mice express lower levels of MHCII in homeostasis [20]. As shown in Fig 5C, the expression of MHCII in resident and migratory cDC is not altered in the LN and SP of the Inhα^-/-^ compared to Inhα^+/+^ mice. Interestingly, LCs in Inhα^-/-^ mice exhibited lower MHCII expression, as assessed by immunofluorescence staining of epidermal sheets and flow cytometry, which was accompanied by increased cell spreading compared to Inhα^+/+^ (Fig 5D).

### Inhα^-/-^ DCs show impaired maturation *in vivo*

**Fig 6:**
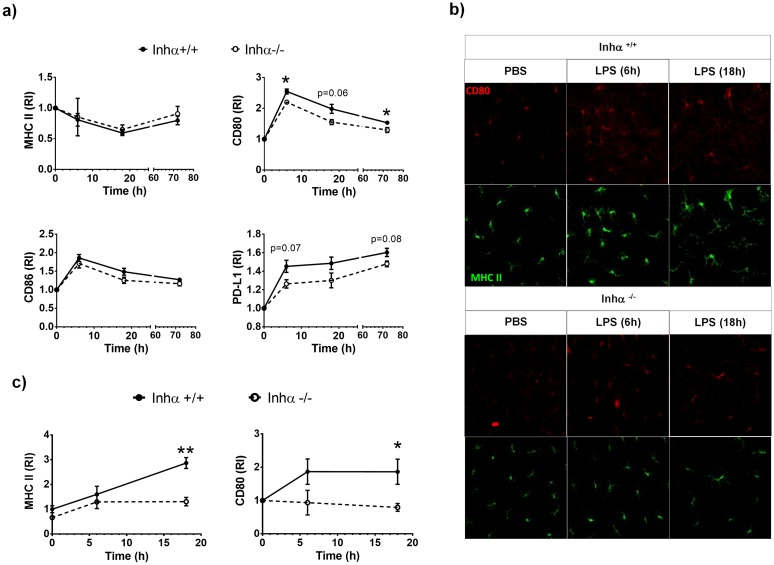
LPS-induced DC maturation *in vivo* is impaired in Inhα^-/-^ mice. Inhα^+/+^ and Inhα^-/-^ 3-week-old mice were inoculated intradermal with LPS (1μg) in left ear and PBS in right ear as control. After 6, 18 and 72 h, dLN and ear epidermal layers were obtained to evaluate DC maturation. A. Time course of MHCII, CD80, CD86 and PD-L1 expression in cDCs (CD11c^+^ MHCII^+^). B. Confocal microscopy micrographs of epidermal sheets stained for MHCII (green) and CD80 (red). C. MHCII and CD80 relative expression in LC from epidermal sheets. Graphs represent relative increment of LPS-ear versus PBS-ear mean ± SEM of 5 independent experiments. Statistical significance was determined by using a two tailed unpaired Student t-Test *p≤0.05; **p≤0.01; ***p≤0.001.

Although no differences were found in DC subpopulations under steady state conditions, we next evaluated the effect of the absence of Inhibins under inflammatory conditions. To analyze DC maturation *in vivo* we inoculated subcutaneously LPS or PBS in the ears of 3-week-old Inhα^-/-^ and Inhα^+/+^ mice, and analyzed expression of maturation markers in DCs from dLN after 6h, 18h and 72 h. As shown in Fig 6A, Inhα^-/-^ cDCs expressed lower levels of CD80 and PD-L1 after LPS inoculation compared to Inhα^+/+^ cDCs. CD86 expression was also slightly decreased in Inhα^-/-^ cDCs compared to Inhα^+/+^, although differences were not statistically significant. As we previously found differences in LC phenotype under steady state conditions, we also evaluated MHCII and CD80 after LPS inoculation in epidermal sheets. As shown in Fig 6B, Inhα^+/+^ LCs increased MHCII and CD80 surface levels as early as 6 hours after LPS inoculation, whereas no changes were observed in LCs from PBS-inoculated ear (Fig 6B and 6C). In contrast, the upregulation of MHCII and CD80 was not observed in LPS-stimulated Inhα^-/-^ LCs, strongly suggesting that, similarly to cDCs, LC maturation is impaired in the absence of Inhibins. To further characterize skin DCs we also used the CD207, CD11b and CD103 markers and analyzed LC, CD11b+, CD11c- and CD103+ subpopulations (S3A Fig) after digestion of skin sheets after 18h of LPS inoculation. As shown in S3B Fig, Inhα^-/-^ LC expressed lower levels of MHCII, and CD80 compared to Inhα^+/+^ mice.

### Impaired DTH responses in Inhα^-/-^ mice

**Fig 7:**
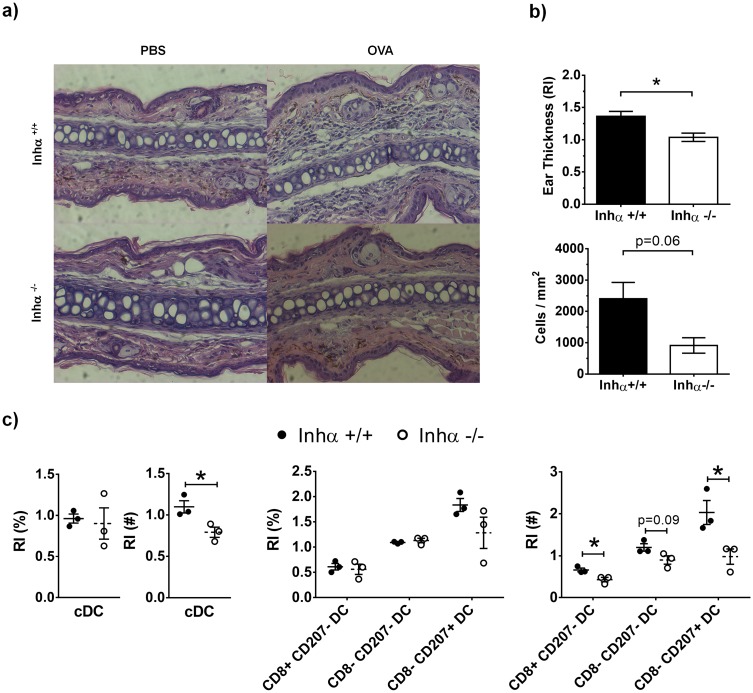
Inhα^-/-^ mice exhibit impaired DTH response induced by OVA challenge. Mice were immunized with 100ng OVA + 10μg LPS in the back. After 7 days mice were challenged with 100ng of OVA in the ear. A. H&E staining of ear sections at 24h after challenge from a representative experiment is shown. B. Ear thickness (top), expressed as relative increment between OVA-ear thickness divided by PBS-ear thickness, and quantification of infiltrating cells (bottom) in challenged ears after 24h. Graphs represent mean ± SEM of 2 independent experiments **(n = 3).** C. Percentages and numbers of cDC subsets in dLN expressed as relative increment between challenged ears divided by PBS ears. Graphs represent mean ± SEM of 2 independent experiments (n **=** 3). Asterisks denote statistical significance *p≤0.05; **p≤0.01; ***p≤0.001.

To investigate whether the impaired maturation of DCs in response to LPS may affect T cell activation, we next performed a DTH assay in response to OVA. Inhα^-/-^ and Inhα^+/+^ mice were inoculated subcutaneously with OVA using LPS as adjuvant (as described in materials and methods). As expected, cells recruited to the ear of Inhα^+/+^ mice challenged with OVA led to ear swelling (1.4 fold compared to PBS) (Fig 7A), while Inhα^-/-^ mice did not show a significant DTH response (Fig 7B). Furthermore, when ear swelling was measured every 12 hours up to 72 hours, Inhα^-/-^ mice showed no inflammation through the time course (S4 Fig). The lack of ear swelling in Inhα^-/-^ mice correlated with a poor cellular infiltration within the ear (Fig 7A). Additionally, when DC subpopulations were evaluated at 24 hours, Inhα^-/-^ mice showed lower numbers of cDCs compared to Inhα^+/+^ mice (Fig 7C). Furthermore, analysis of DC subpopulations, showed that the CD8α^-^ CD207^+^ DC subpopulation, which is composed mainly by LC, was the most reduced subpopulation in dLN from Inhα^-/-^ mice after OVA challenge. Our data suggest that the lack of DHT responses in Inhα^-/-^ mice may be due to a combination of impaired DC maturation and migration.

## Discussion

There is a growing evidence of the influence of Activins in the regulation of immunity, however, little is known about the role of Inhibins in these processes. Our group has previously demonstrated that Inhibins are required for DC maturation in the thymus and that in the absence of Inhibins, reduced levels of MHCII and CD86 on stromal DCs have an impact in thymocyte selection [12]. However, the role of Inhibins in DC maturation and function in the periphery had not been explored. Our results showed that the absence of Inhibins results in impaired BMDC maturation *in vitro*, evidenced by reduced expression of MHCII, CD80 and CD86, and increased levels of the co-inhibitory molecule PD-L1 in response to LPS. The low expression of MHCII and CD86 contradicts previously reported data showing that Inhibin A is capable of preventing the upregulation of HLA-DR expression during human Mo-DC maturation *in vitro* in response to pro-inflammatory cytokines, including as Protaglandin E2, IL-1β, IL-6 and TNFα [27]. However, it is worth noting that Activin ligands may exert different effects on immune cells, depending on the developmental stage, under inflammatory [32,33] or steady state conditions [34] and in response to different stimuli [28].

Unexpectedly, CCR7 expression on mBMDC was not affected in the absence of Inhibins (Fig 2C), despite their reduced capacity to migrate towards CCL19 and CCL21 *in vitro* and their impaired homing to the LN *in vivo* (Fig 2A and 2B), which may imply alterations in CCR7-dependent signaling. Alternatively, the morphological alterations of mBMDC from Inhα^-/-^mice suggest possible differences in the expression and/or function of adhesion molecules. In an attempt to investigate the potential defects in CCR7 mediated signaling, Ca^++^ fluxes and Erk phosphorylation in response to chemokines CCL19 and CCL21 were evaluated. Although Ca^++^ mobilization was not significantly different between Inhα^+/+^ and Inhα^-/-^ mBMDC, Erk phosphorylation was enhanced in Inhα^-/-^ mice compared to Inhα^+/+^ (Fig 2). The functional relevance of this enhanced Erk phosphorylation remains unknown, however we cannot rule the possibility of a disbalance in signaling pathways downstream CCR7 being involved in the impaired cell migration. In this context, we previously reported that the impaired migration of Jak 3^-/-^ T lymphocytes, correlated with an hyperactivation of cofilin, which is a target of Erk, resulting in altered actin cytoskeleton dynamics[35].

The altered phenotype displayed by Inhα^-/-^ DCs suggested that they might also be impaired in their functions. Indeed, our data demonstrated that Inhα^-/-^ mBMDCs induced poor allogeneic T cell responses, possibly as a consequence of lower levels of MHCII and CD86 expression and increased PD-L1 expression. In this context, PD-L1 has been shown to down regulate T cell receptor (TCR) signaling by recruiting SHP-2 [36] and consequently, promotes T cell anergy and Treg induction [37,38]. Whether the impaired maturation of Inhα^-/-^ BMDCs has an impact on T cell polarization and Treg induction remains unclear and is currently being investigated.

A potential mechanism involved in the altered DC maturation observed in the absence of Inhibins may be the overproduction of Activins. Accordingly, the absence of α-subunits would result in enhanced β-dimer assembly (over αβ heterodimer formation) leading to increased Activin expression [14]. In this context, Robson et al. previously demonstrated that Mo-DCs secrete Activin A in response to TLR ligands (LPS, R-848, Poly I:C and Pam3Cys), CD40L and Prostaglandin E2, acting as an autocrine negative feed-back mechanism to counteract DC maturation [28]. Interestingly, no significant differences were observed in Activin A secretion between Inhα^+/+^ and Inhα^-/-^ BMDCs after LPS stimulation (Fig 4B), arguing against the involvement of Activin A in the altered maturation of Inhα^-/-^ BMDCs. Hence, although it was previously reported that recombinant Activin A and Inhibin A can down regulate DC maturation mediated by pro-inflammatory cytokines [27], our data indicate that Inhibins promote DC maturation in response to LPS.

Beyond the role of Inhibins as antagonists of Activin signaling, evidence has accumulated supporting a possible mechanism underlying Inhibin-mediated functions [9]. Indeed, there are reports suggesting that Inhibins may transduce signals through an independent receptor (reviewed in [10]). Therefore, Inhibins may have their own role in the regulation of DC maturation. Our results demonstrate that BMDC can secrete significant levels of Inhibin A upon LPS stimulation, showing a peak of secretion at 18h. Interestingly, at this time point, Inhα^-/-^ BMDC produced higher levels of IL-10 and slightly lower levels of IL-6 compared to Inhα^+/+^ BMDCs (Fig 4), which may partially account for reduced stimulatory capacity of these cells (Fig 3). However, Inhibin mediated signals do not appear to directly regulate cytokine production in response the fact that exogenous addition of Inhibin A was unable to restore IL-10 and IL-6 levels Inhα^-/-^ BMDC in response to LPS, suggests that Inhibins may modulate DC maturation through other mechanisms. In this context, recombinant Inhibin A was able to restore MHCII upregulation in Inhα^-/-^ BMDC when added at the time of LPS stimulation (data not shown).

The enhanced production of IL-10 and slightly reduced levels of IL-6 in Inhα^-/-^ BMDC might imply the activation of different signaling pathways downstream TLR stimulation. In this context, TLR stimulation leads the expression of NFκB-dependent pro-inflammatory cytokines, but also activates MAPK-dependent transcription factors AP-1 and CREB [39]. It has been shown that IL-10 is produced in a CREB-dependent manner in macrophages after TLR2/TLR4 activation, downstream the phosphorylation of p38 and other MAPKs [40]. On the other hand, in multiple myeloma, IL-6 production is down regulated by CREB binding to the CREB site within the IL-6 promoter region, independently of the NFκB [41]. Therefore, one possible mechanism to explain the enhanced IL-10 and decreased IL-6 production by Inhα^-/-^ BMDC might involve preferential activation of CREB over AP-1 in response to LPS.

Another cytokine that has been shown to down regulate DC maturation is TGFβ. Indeed, DCs acquire a tolerogenic phenotype in the presence of TGFβ, characterized by low expression of DC maturation markers such as CD83, CD80 and CD86, as well as MHCII, and down regulation of pro-inflammatory cytokines, such as TNFα, IL-12 and IFNα, with a promotion of regulatory cytokines release, such as TGFβ [5].

Although in the absence of Inhibins mBMDC displayed a “semi-mature” phenotype, we were unable to detect significant levels of TGFβ in the supernatant of LPS stimulated cultures (data not shown), arguing against the possibility of this cytokine playing a role.

Whether Inhibins play a role in DC differentiation *in vivo* remains unclear. Our results show that *in vitro* DC differentiation from bone marrow progenitors is significantly diminished in the absence of Inhibins (Fig 1A), however no differences were found in the numbers or percentage of cDC in LN and spleen of Inhα^-/-^ mice compared to Inhα^+/+^ mice (Fig 5A). This discrepancy might be explained by the fact that *in vivo* arriving pre-DCs may be provided with a tissue-specific cytokine milieu that may compensate the DC generation in the absence of Inhibins (reviewed in [42,43]).

There is evidence showing that members of TGFβ superfamily promote LC differentiation in an autocrine manner [44]. In this context, Musso et al. demonstrated that transgenic overexpression of Activin A under the keratin promoter induced enhanced LC differentiation from circulating monocytes, while Follistatin (ligand trap for Activins), reduced their generation *in vivo* [30]. However, serum Activin A levels were not significantly different in 3 week-old Inhα^-/-^ mice compared to Inhα^+/+^ (S2A Fig). Interestingly, although LC numbers in epidermal sheets were not altered compared to Inhα^+/+^mice, LC phenotype was altered, showing decreased MHCII levels and increased cell spreading, suggesting a role for Inhibins in LC homeostasis. Under inflammatory conditions, LC increase MHCII and co-stimulatory molecules and, in parallel, lose their extended phenotype and acquire a round shape, that allows them to migrate to LN. These changes are accompanied by down regulation of E-cadherin and up-regulation of N-cadherin and CCR7 (reviewed in [45]). Interestingly, upregulation of costimulatory markers on LC was impaired after *in vivo* LPS stimulation in Inhα^-/-^ mice, which correlates with the *in vitro* results of BMDC maturation. In addition, Inhα^-/-^ LC also displayed differences in morphology compared to Inhα^+/+^, which may indicate an increase in adhesion molecule expression, such as E-cadherin.

Finally, to exert an immune response *in vivo*, DCs capture antigens in the periphery and migrate to dLN in order to present them and activate T cells. In DTH assays, both LC and dermal DCs are important to induce a T cell response [45]. The Inhα^-/-^ mice showed poor DTH in response to OVA challenge, which could be the result of a decreased maturation and consequently reduced migration of CD8^-^ CD207^+^ DCs to the dLN. As this local immune response is dependent on skin DCs, it still remains to be evaluated the effect inhibin-mediated functions in systemic immune responses or in specific tolerogenic microenvironments, such as the intestinal tract, which may require the participation of other DC subpopulations.

In summary, here we provide the first evidence that Inhibins play a key role in promoting DC maturation and migration potentiating their function to induce T cell responses. Our data suggest that these ligands could be considered as potential targets to down modulate immune responses in specific inflammatory conditions.

## Supporting Information

S1 Fig *In vitro* differentiation, analysis of apoptosis and TLR4 expression in Inhα^-/-^ or Inhα^+/+^ BMDC. A. Total cell numbers at day 6 of BMDC culture (7 independent experiments). B. Percentage of Anexin V^+^, Anexin V^+^ 7AAD^+^ and 7AAD^+^ of DC, CD11c^+^ MHCII^-^ and CD11c^-^ analyzed by flow cytometry. (5 independent experiments). C) Representative confocal micrographs (100x) from iBMDCs and mBMDCs stained with phalloidin rhodamine from 3 individual Inhα^+/+^ (left) and Inhα^-/-^ (right) mice, respectively, before and after LPS treatment. D). TLR4 expression gated on CD11c^+^ MHCII^+^ population analyzed by flow cytometry. MFI values are shown and expressed as mean ± SEM of 4 independent experiments. E) Analysis of MHCII upregulation in response to different maturation stimuli at 24h. Data are expressed as relative expression compared to iDC expression. Mean± SEM of 3 independent experiment (n = 5–6 mice).
(TIF)

S2 Fig Analysis of serum Activin and e*x vivo* analysis of DC subpopulations. A. Activin A was measured in Inhα^+/+^ and Inhα^-/-^ 3 week-old-mice by ELISA. Graph represents at least 3 independent mice. B-C. Gating strategy to define DC subsets in LN (B) and spleen (C) of Inhα^+/+^ and Inhα^-/-^ mice. B. Within the cells suspensions, CD19^-^ CD3^-^ TER119^-^ NK1.1^-^ single live cells were selected for further analysis. The CD11c^hi^ MHC II^Int^ population represents lymphoid resident DCs, and can be further divided into CD8α^+^ and CD8α ^-^ DC. CD11c^Int^ MHC II^hi^ population represents migratory DCs, that can be further divided into CD11b-, CD11b+ CD103^+^ and LC (CD207+, CD11b+, CD103-). C. Splenic CD19^-^ CD3^-^ TER119^-^ NK1.1^-^ single live cells were selected. The CD11c^hi^ MHC II^Int^ population represents spleen resident DC, and can be further divided into CD8^+^ and CD8^-^ DCs.
(TIF)

S3 Fig Skin DCs display impaired maturation *in vivo*. Inhα^+/+^ and Inhα^-/-^ 3-week-old mice were inoculated intradermal with LPS (1μg) in left ear and PBS in right ear as control. After 18h, ears were digested to evaluate skin DC subset maturation. A. Gating strategy to define DC subsets. The CD11c^+^ MHC II^+^ population represents cDCs and can be further divided into four subpopulations based on the expression of CD11b, CD207 and CD103 markers. B. Analysis of DC subpopulations from digested skin sheets. Expression of MHCII, CD80, PD-L1 and CCR7 after 18h of LPS stimulation in CD11b-, CD11b+, CD103+ and LC (n = 2).
(TIF)

S4 Fig Impaired DTH response in Inhα^-/-^ mice. Mice were immunized with 100ng OVA + 10μg LPS in the back. After 7 days, mice were challenged with 100ng of OVA in one ear and PBS in the other ear for control. Ear thickness was measured through the time for up to 72 hours. Graphs represent mean ± SEM of 2 independent experiments.
(TIF)
