# Supplementary figures and images for: A Key Role for Inhibins in Dendritic Cell Maturation and Function
Source: PLoS One. 2016 Dec 9;11(12):e0167813. doi: 10.1371/journal.pone.0167813 (PMC5147992; doi:10.1371/journal.pone.0167813)

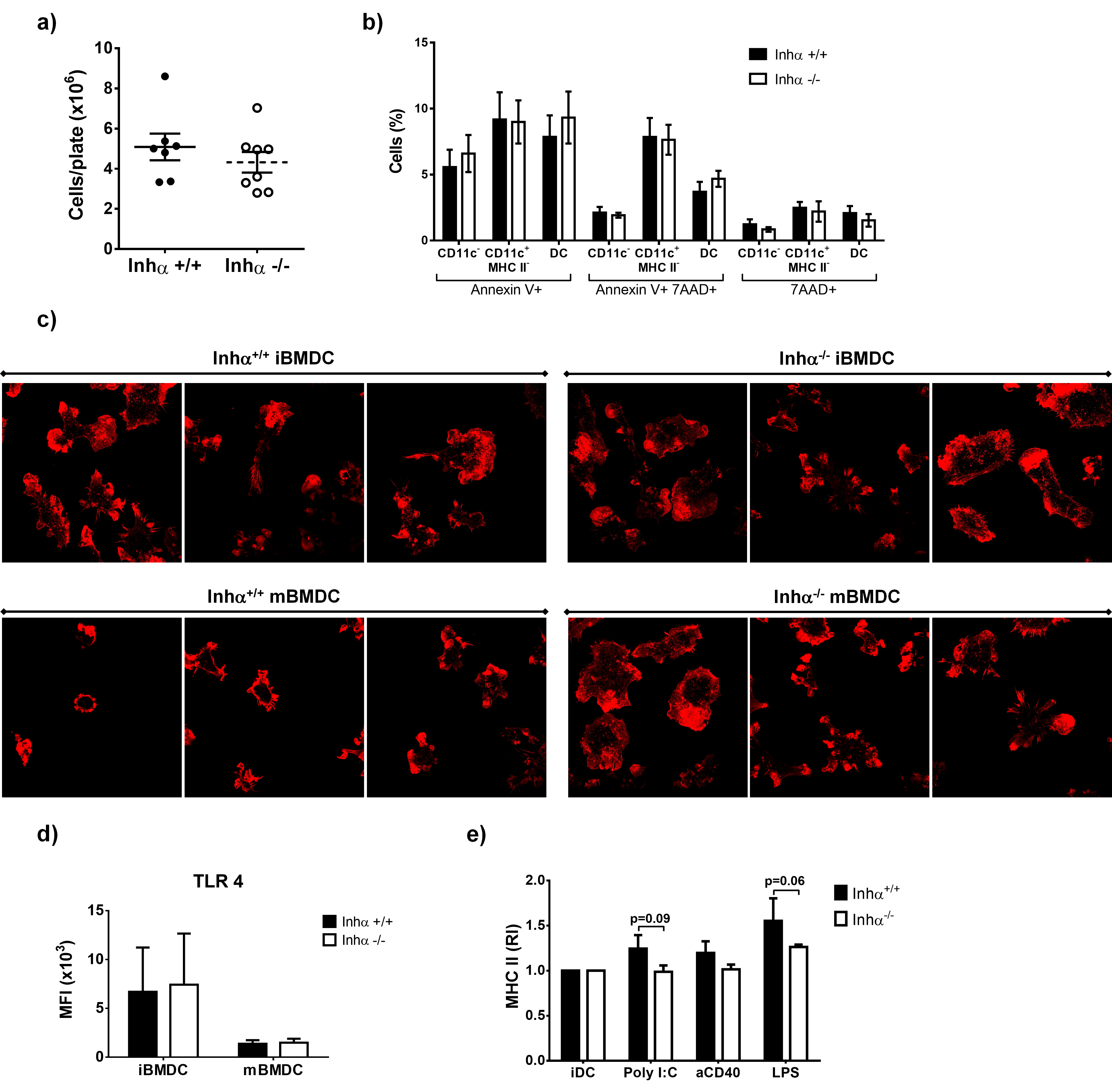

Supplement: S1 Fig — A. Total cell numbers at day 6 of BMDC culture (7 independent experiments). B. Percentage of Anexin V+, Anexin V+ 7AAD+ and 7AAD+ of DC, CD11c+ MHCII- and CD11c- analyzed by flow cytometry. (5 independent experiments). C) Representative confocal micrographs (100x) from iBMDCs and mBMDCs stained with phalloidin rhodamine from 3 individual Inhα+/+ (left) and Inhα-/- (right) mice, respectively, before and after LPS treatment. D). TLR4 expression gated on CD11c+ MHCII+ population analyzed by flow cytometry. MFI values are shown and expressed as mean ± SEM of 4 independent experiments. E) Analysis of MHCII upregulation in response to different maturation stimuli at 24h. Data are expressed as relative expression compared to iDC expression. Mean± SEM of 3 independent experiment (n = 5–6 mice). (TIF) [file pone.0167813.s001.tif]

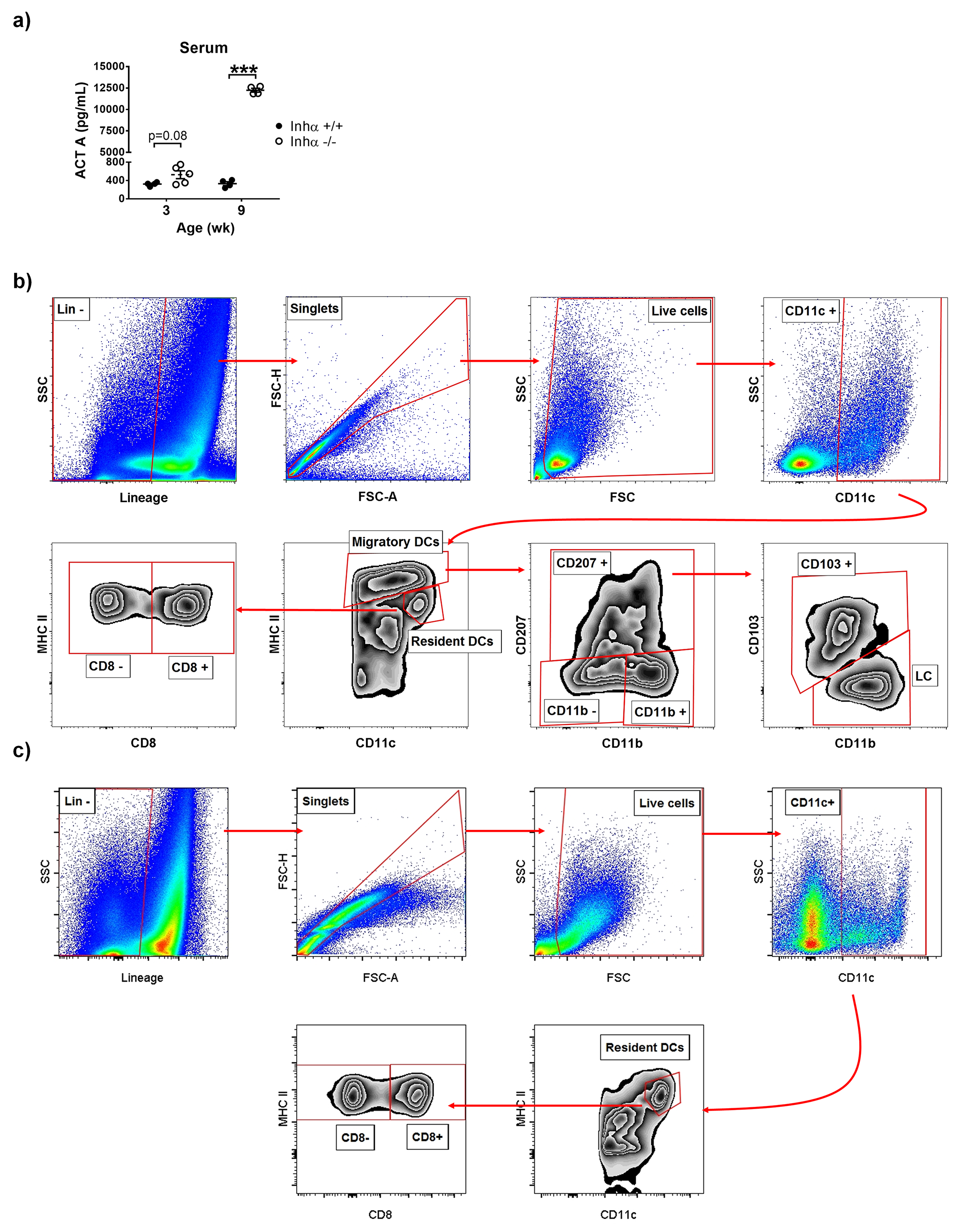

Supplement: S2 Fig — A. Activin A was measured in Inhα+/+ and Inhα-/- 3 week-old-mice by ELISA. Graph represents at least 3 independent mice. B-C. Gating strategy to define DC subsets in LN (B) and spleen (C) of Inhα+/+ and Inhα-/- mice. B. Within the cells suspensions, CD19- CD3- TER119- NK1.1- single live cells were selected for further analysis. The CD11chi MHC IIInt population represents lymphoid resident DCs, and can be further divided into CD8α+ and CD8α - DC. CD11cInt MHC IIhi population represents migratory DCs, that can be further divided into CD11b-, CD11b+ CD103+ and LC (CD207+, CD11b+, CD103-). C. Splenic CD19- CD3- TER119- NK1.1- single live cells were selected. The CD11chi MHC IIInt population represents spleen resident DC, and can be further divided into CD8+ and CD8- DCs. (TIF) [file pone.0167813.s002.tif]

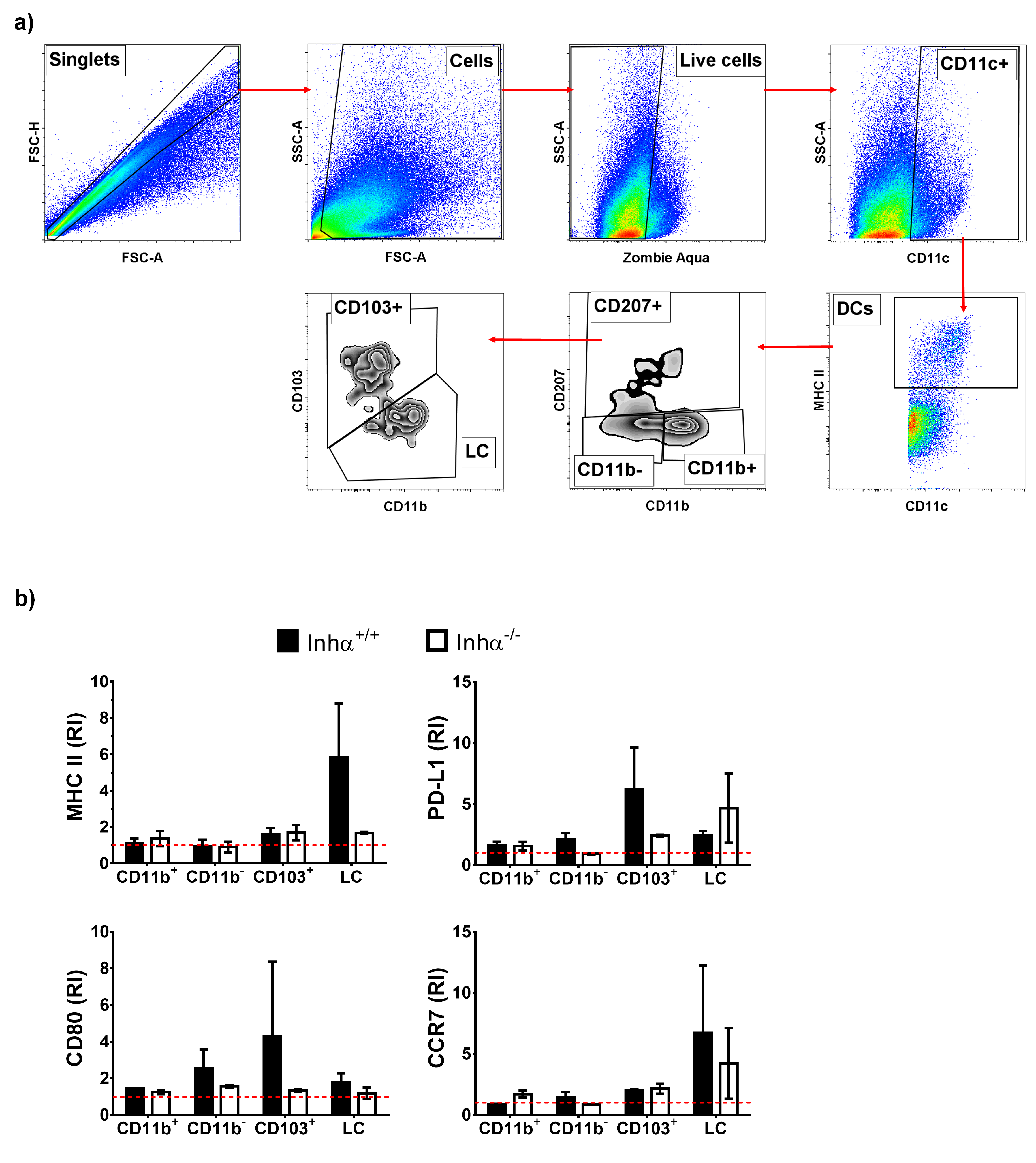

Supplement: S3 Fig — Inhα+/+ and Inhα-/- 3-week-old mice were inoculated intradermal with LPS (1μg) in left ear and PBS in right ear as control. After 18h, ears were digested to evaluate skin DC subset maturation. A. Gating strategy to define DC subsets. The CD11c+ MHC II+ population represents cDCs and can be further divided into four subpopulations based on the expression of CD11b, CD207 and CD103 markers. B. Analysis of DC subpopulations from digested skin sheets. Expression of MHCII, CD80, PD-L1 and CCR7 after 18h of LPS stimulation in CD11b-, CD11b+, CD103+ and LC (n = 2). (TIF) [file pone.0167813.s003.tif]

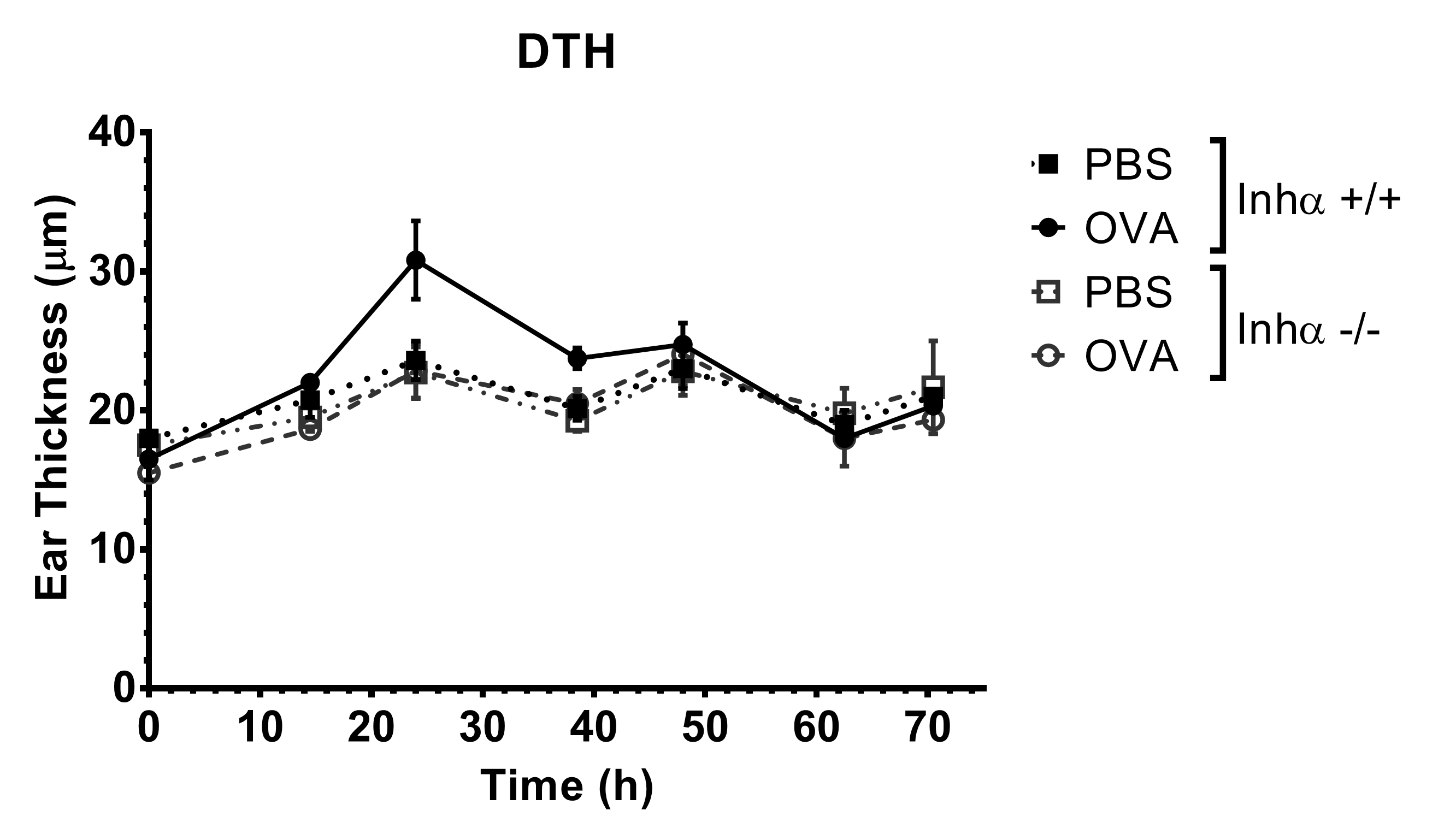

Supplement: S4 Fig — Mice were immunized with 100ng OVA + 10μg LPS in the back. After 7 days, mice were challenged with 100ng of OVA in one ear and PBS in the other ear for control. Ear thickness was measured through the time for up to 72 hours. Graphs represent mean ± SEM of 2 independent experiments. (TIF) [file pone.0167813.s004.tif]
